# Lynx Prototoxins: Roles of Endogenous Mammalian Neurotoxin-Like Proteins in Modulating Nicotinic Acetylcholine Receptor Function to Influence Complex Biological Processes

**DOI:** 10.3389/fphar.2019.00343

**Published:** 2019-04-30

**Authors:** Julie M. Miwa, Kristin R. Anderson, Katie M. Hoffman

**Affiliations:** Department of Biological Sciences, Lehigh University, Bethlehem, PA, United States

**Keywords:** nicotine, toxins, cholinergic modulation, learning, plasticity

## Abstract

The cholinergic system modulates many biological functions, due to the widespread distribution of cholinergic neuronal terminals, and the diffuse release of its neurotransmitter, acetylcholine. Several layers of regulation help to refine and control the scope of this excitatory neurotransmitter system. One such regulatory mechanism is imparted through endogenous toxin-like proteins, prototoxins, which largely control the function of nicotinic receptors of the cholinergic system. Prototoxins and neurotoxins share the distinct three finger toxin fold, highly effective as a receptor binding protein, and the former are expressed in the mammalian brain, immune system, epithelium, etc. Prototoxins and elapid snake neurotoxins appear to be related through gene duplication and divergence from a common ancestral gene. Protein modulators can provide a graded response of the cholinergic system, and within the brain, stabilize neural circuitry through direct interaction with nicotinic receptors. Understanding the roles of each prototoxin (e.g., lynx1, lynx2/lypd1, PSCA, SLURP1, SLURP2, Lypd6, lypd6b, lypdg6e, PATE-M, PATE-B, etc.), their binding specificity and unique expression profile, has the potential to uncover many fascinating cholinergic-dependent mechanisms in the brain. Each family member can provide a spatially restricted level of control over nAChR function based on its expression in the brain. Due to the difficulty in the pharmacological targeting of nicotinic receptors in the brain as a result of widespread expression patterns and similarities in receptor sequences, unique interfaces between prototoxin and nicotinic receptor could provide more specific targeting than nicotinic receptors alone. As such, this family is intriguing from a long-term therapeutic perspective.

## Introduction

The highly successful toxin fold structure found in many venomous snake toxins also has a counterpart in mammals: prototoxins. Prototoxins are non-venomous proteins with significant similarities to elapid α-neurotoxins, most notably with regard to their cysteine-rich, three-fingered β-fold structure. The mammalian counterparts have 10–12 cysteine residues and the signature motif of 10 cysteines participating in five disulfide bonds stabilizing a β-fold structure. Prototoxins have been found in the brain, epithelium, and immune system, etc. (reviewed in Miwa et al., 2011; Tsetlin, 2015; Vasilyeva et al., 2017). Within the prototoxin family, there are membrane-bound GPI anchored (i.e., lynx1, lynx2, PSCA, etc.) and secreted forms of the protein (i.e., secreted ly6/uPAR related proteins SLURPs) (Yan et al., 1998; Adermann et al., 1999).

Snake toxins employ functional mimicry of pathways operating in the prey. The cloning of a cDNA from the mammalian brain (Kuhar et al., 1993) with the cysteine-rich signature of α-neurotoxins, was suggestive that it acted on a similar molecular target as α-neurotoxins (Miwa et al., 1999). Although orphan members of the mammalian superfamily had been previously identified (Ploug and Ellis, 1994), the understanding that toxins employ functional mimicry of pathways operating in the prey led to a candidate approach for investigating their function, ultimately resulting in the discovery of the initial functionally characterized prototoxin with nicotinic receptor modulatory capability, lynx1 (Miwa et al., 1999; Ibanez-Tallon et al., 2002).

Current evidence suggests snake toxins arose through gene duplication and divergence from a prototoxin-like ancestral gene (Adermann et al., 1999). The presence of these two forms of prototoxins, GPI and secreted, could provide clues to understanding the evolutionary relationship of venoms and prototoxins.

In the secreted SLURP genes, a stop codon occurs prior to genomic sequence which could code for the amino acid consensus sequence for GPI-attachment, suggests that the membrane-bound forms evolved prior to the secreted forms [i.e., secreted ly6/uPAR-related proteins (SLURPs)] (Yan et al., 1998; Adermann et al., 1999). Loss of the GPI anchor via introduction of a stop-gain mutation to generate a secreted version of the protein may be an important intermediate step in the progression from an ancestral prototoxin to the first α-neurotoxin. On the other hand, evidence of accelerated evolution in the membrane-bound form may support that notion that the GPI-anchored version evolved more recently (Dorus et al., 2004). Regardless, the three-fingered fold toxin-like proteins seems to have occurred once, prior to the split between the venomous elapid snakes and the non-venomous colubrid snake family. This is supported by previous research which isolated and characterized three-fingered toxins from non-venomous colubrid snakes, demonstrating that this toxin type exhibits basal α-neurotoxic activity (Fry et al., 2003a). This evidence suggests that gene recruitment occurred via a non-toxic gene in the body (Fry and Wüster, 2004). Venom development requires a transition of genes to expression in the secretory venom gland of the snake (Kessler et al., 2017), either via recruitment from a gene in the body (Fry, 2005) or restricted expression of a gene with wider expression, such as those in the salivary glands (Reyes-Velasco et al., 2015).

**FIGURE 1:**
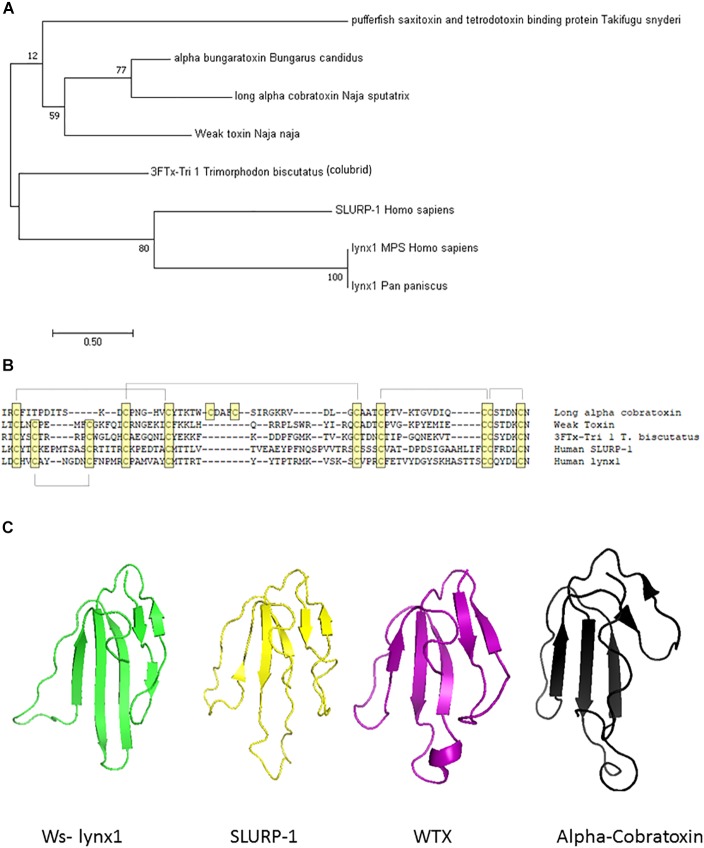
Molecular, phylogenetic, and structural analysis of the prototoxin gene family. **(A)** Bootstrap consensus tree of molecular evolutionary relationship. The percentage of replicate trees in which the associated taxa clustered together in the bootstrap test (500 replicates) are shown next to the branches. **(B)** Amino acid comparison of selected family members. Cysteines are highlighted in yellow and disulfide bridges are outlined. **(C)** Structural comparison of selected family members from the pdb database: Ws-lynx1 (2L03, green), SLURP-1 (2MUO, yellow), Weak toxin (2MJO, purple), Cobratoxin (2CTX, black).

A few three-fingered fold protein family members have been reported to exhibit expression in the salivary gland of non-venomous species, providing possibilities for more neutral selection (Hijazi et al., 2009). More in-depth genomic and transcriptomic analyses of the non-venomous family will be required as more data on the snake genome become available. Although other genes, such as digestive track enzymes or ribonucleases (Strydom, 1973), have been proposed as the ancestral gene giving rise to α-neurotoxins, separate recruitment events seem to be involved (Fry, 2005). Gene duplication aids in the development of venoms, as it allows for divergence in either amino acid sequence to more virulent forms or in regulatory elements to allow such changes in gene expression patterns. There is substantial evidence for gene duplication within mammalian prototoxins/non-venomous species allowing for such sub-functionalization, although the presence of clustered ly6 genes in mice, but not in humans, suggests that at least some of this duplication occurred relatively recently after the divergence of mice and humans (Loughner et al., 2016). Furthermore, snake toxins have the ability to undergo accelerated evolution and selective expression in the snake venom gland (Fry et al., 2003b; Kessler et al., 2017). The evolutionary relationships between members of the prototoxin gene family, WTX, venomous snakes, and colubrid family members are depicted in Figure 1.

## Introducing the Prototoxin Gene Family

The prototoxin genes are members of the ly6/uPAR superfamily, whose members adopt the receptor binding structural motif observed within elapid snake venom α-neurotoxins, due to highly conserved cysteine residues that participate in disulfide bonding (Lyukmanova et al., 2011; Tsetlin, 2015; Vasilyeva et al., 2017). Among these family members are the well-studied single-domain snake venom α-neurotoxins and cardiotoxins (Fleming et al., 1993; Ploug and Ellis, 1994). Three-fingered proteins are larger and generally more variable than α-neurotoxins (Kessler et al., 2017). Although α-bungarotoxin (α-btx) is one of the most widely used snake toxins in neuroscience, exhibiting nearly irreversible affinity, ly6/uPAR family members have shown variability in their ability to compete with other nAChR ligands, suggesting more modest receptor affinities and potentially allosteric effects on receptor function. The expression of three-fingered proteins in the brain and body allows for biological regulation over complex nicotinic receptor-based processes across multiple systems. Members of the uPAR superfamily include CD59, lymphocyte antigen genes, ly6A-H, transforming growth factor β receptor ectodomains, and uPAR. In total, at least 2,583 sequences within seven subfamilies have been identified (PFAM database) (Kessler et al., 2017). Further, the human genome encodes for at least 45 genes containing the three-fingered domain (Galat, 2008). Three-fingered proteins exert an influence over a wide-array of physiological processes, including proliferation, cellular differentiation, and inflammation, among others. The present review focuses on members of this family with demonstrated nAChR modulatory/binding function, with special emphasis on those expressed in the mammalian brain. Within this large superfamily, members with significant expression in the brain include lynx1 (Miwa et al., 1999), lynx2/lypd1 (Dessaud et al., 2006; Wu et al., 2015), lypd6 (Darvas et al., 2009; Zhang et al., 2010), lypd6B (Demars and Morishita, 2014), PSCA (Jensen et al., 2015), and ly6h (Horie et al., 1998). SLURPs, which are expressed mostly outside the brain, have been documented in several disease states and will be also be reviewed (Grando, 2008; Vasilyeva et al., 2017).

## Structural Features of the Prototoxin and ly6/uPar Superfamily

**FIGURE 2:**
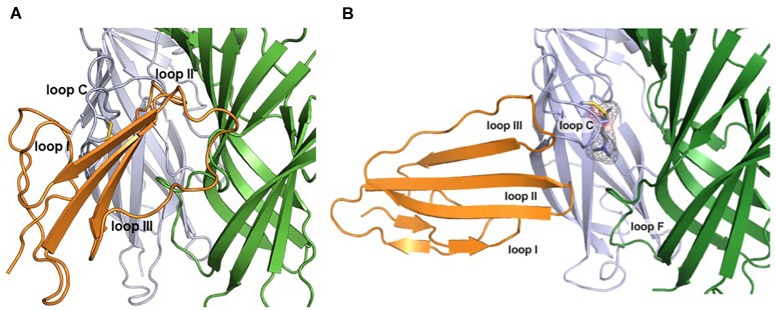
Computational models of lynx1 interaction with nicotinic receptor subunits. **(A)** Co-model of ws-lynx1 and α4: α4 nAChR interface (Nissen et al., 2018). **(B)** Co-model of ws-lynx1 and α7 nAChRs (Lyukmanova et al., 2011).

Prototoxins share structural and functional similarities to one another. Among their most notable structural features are cysteine bonds that stabilize a three-loop/three-fingered β-rich fold structure. The ly6/uPAR superfamily contains 10–12 cysteines, with one of the extra disulfide bonds in the first loop, placing this mammalian family closer to the weak toxins depicted in Figure 1. NMR solution structures have greatly enhanced our understanding of this class of proteins (Lyukmanova et al., 2011, 2014, 2016b; Paramonov et al., 2017). A recombinant version of lynx1 was engineered such that the sequence for GPI anchor attachment sequence was removed, water soluble lynx1 (ws-lynx1) (Lyukmanova et al., 2011). The NMR solution structure of ws-lynx1 demonstrated that the overall topology is similar to that of α-neurotoxins, although the protein is characterized by a large and mostly disordered loop III (Lyukmanova et al., 2011). The addition of a disulfide bond in loop I is common among other prototoxins and weak/unconventional snake toxins. NMR evaluation was performed for SLURP-1, synthetically derived using peptide ligation (sSLURP-1), and exhibits excellent agreement to that of rSLURP-1 in two- and three-dimensional spectral analyses (Durek et al., 2017). Unfortunately, to date, few crystal structures of prototoxins have been solved, although computational models have been developed for prototoxins and their cognate receptors (Figure 2). Solution and crystal structures have been identified for members of the larger superfamily, including CD59 (Fletcher et al., 1994; Huang et al., 2007). CD59 as well as lypd6 and lypd6b (Paramonov et al., 2017) contains an α-helical domain in loop III, which is not found in the NMR structures of lynx1 (Lyukmanova et al., 2011), underscoring the notion that there is more structural variability among the three-fingered proteins of the superfamily than within three-fingered α-neurotoxins (Kessler et al., 2017).

## The Cholinergic System

The cholinergic system is a widespread modulatory excitatory neurotransmitter system that enables controlled regulation over multiple neural circuits. Cholinergic neurons release the endogenous neurotransmitter acetylcholine in a diffuse manner. Cholinergic projection neurons are located in the basal forebrain and brain stem, and their terminals radiate broadly throughout the central nervous system (Guo et al., 2015) onto a wide range of targets. This widespread architecture and the fact that neurotransmitter release is not confined to the synaptic cleft contribute to the modulatory capabilities of the cholinergic system. Interestingly, the activity-response relationship of the cholinergic system falls along an inverted U-shaped curve, whereby both impaired and excessive activation can exert deleterious or suboptimal effects (Picciotto, 2003). The cholinergic system operates along a gradient, with detrimental effects observed at the extremes of the range, and is therefore dependent on several regulatory mechanisms (Miwa et al., 2012).

Such fine-tuning of the cholinergic system can be exerted by several factors, including the number and activity of cholinergic neurons, the level of acetylcholine release, the presence of acetylcholinesterase, and the profile of the target receptors. Cholinergic receptors can be divided into muscarinic and nicotinic receptors, which are located mainly in the peripheral and central nervous systems (PNS and CNS), respectively. In addition to acetylcholine, nicotinic receptors bind the exogenous drug of abuse, nicotine. Nicotinic AChRs exist as pentamers composed of many variations of 15 possible subunits. For instance, nicotinic receptors typically exist as heteromeric combinations of α (2–10) and β (2–4) subunits (most commonly α4β2) or as α homopentamers (α7, α9, etc.) (Picciotto, 2003; Albuquerque et al., 2009). Receptor composition gives rise to specificity, as each combination displays distinct biophysical and pharmacological properties. In addition, differences in stoichiometry among subunits allow for differential response profiles and sensitivity to agonist.

Prototoxin proteins can form stable associations with nicotinic receptors, and the binding preference of prototoxin proteins for specific subtypes of nAChRs can further fine tune cholinergic activity by altering a selective subtype or group of receptor subtypes. Further spatial control can be imparted because prototoxins exhibit mostly non-overlapping expression patterns in the CNS (Miwa et al., 2012). Indeed, multifactorial mechanisms such as receptor assembly, expression, and binding specificity contribute to the wide variety of reported effects for each family member (Miwa et al., 2011).

## Lynx1

### Lynx1 Binding and Insights Using Recombinant ws-lynx1

Previous studies have revealed that a recombinant, water-soluble variant of lynx1, ws-lynx1, can inhibit α7, α4β2, and α3β2, although the functional effects are most pronounced for α4β2. The inhibitory effect is concentration-specific and non-competitive (Lyukmanova et al., 2011). Residues on loops II and III are important for the interaction (Lyukmanova et al., 2013). Although recombinant ws-lynx1 studies have been highly informative, the effects may differ from those of endogenous lynx1, since lynx1 is usually attached to the cell surface via a GPI anchor. Researchers have observed differential biological effects between cerebellum-directed transgenic mice expressing a soluble version of lynx1 (minus the amino acid sequence directing the GPI attachment) which leads to augmentations in motor learning, and those expressing normal GPI-bound lynx1 which demonstrates no effect (Miwa et al., 2012). This is in accordance with the differential effects *in vitro* of ws-lynx1 which enhances ACh-evoked current amplitude vs. GPI-linked endogenous lynx1 which causes acceleration of desensitization and lowering of agonist affinity (Miwa et al., 1999; Ibanez-Tallon et al., 2002). The GPI-anchor exhibits an affinity for cholesterol-rich domains (Lester et al., 2012), and the effective concentration (EC_50_) may be higher for the membrane-bound form of lynx1.

### Mechanisms Underlying the Effects of Lynx1 on Receptor Function

Lynx1 exerts its modulatory effect on the cholinergic system via direct interactions with nAChR (Ibanez-Tallon et al., 2002; Nichols et al., 2014). The effects of this interaction on receptor function are multi-factorial, influencing agonist affinity, desensitization, and recovery from desensitization. In *in vitro* studies involving *Xenopus* oocytes, cells co-expressing α4β2 nicotinic receptors and lynx1 (Ibanez-Tallon et al., 2002) demonstrate reduced agonist sensitivity via co-expression of lynx1, as indicated by a rightward shift in the EC_50_ to acetylcholine (Ibanez-Tallon et al., 2002). Furthermore, nAChRs exhibit a faster rate of desensitization to agonists when co-expressed with lynx1, and prolonged recovery from desensitization as assessed by dual application of agonists (Ibanez-Tallon et al., 2002). This finding is in contrast to those of some previous reports (Miwa et al., 1999), which indicated that exogenous application of lynx1 protein to oocytes expressing α4β2 nAChRs increases the amplitude of ACh currents recorded in two-electrode voltage clamp mode.

### Effects of lynx1 on Nicotinic Receptor Assembly

Single-channel activity in α4β2 exhibits a shift toward the expression of high-conductance events and short channel open times (Ibanez-Tallon et al., 2002). This phenotype is associated with the low-sensitivity (LS) (α4)_3_(β2)_2_ stoichiometry. Preferential interaction of lynx with α4: α4 dimers over β2:β2 dimers in the endoplasmic reticulum can help to explain the expression of mature pentamers at the plasma membrane of the LS stoichiometry (α4)_3_(β2)_2_ over the high sensitivity, HS (α4)_2_(β2)_3_ stoichiometry (Nichols et al., 2014). Co-expression studies can be influenced by stoichiometry and assembly, as well as gating activity of nAChRs at the neuronal cell surface of the plasma membrane. It can be difficult to discern the relative contributions of these two effects without cleaving off the GPI anchor via PI-PLC.

**FIGURE 3:**
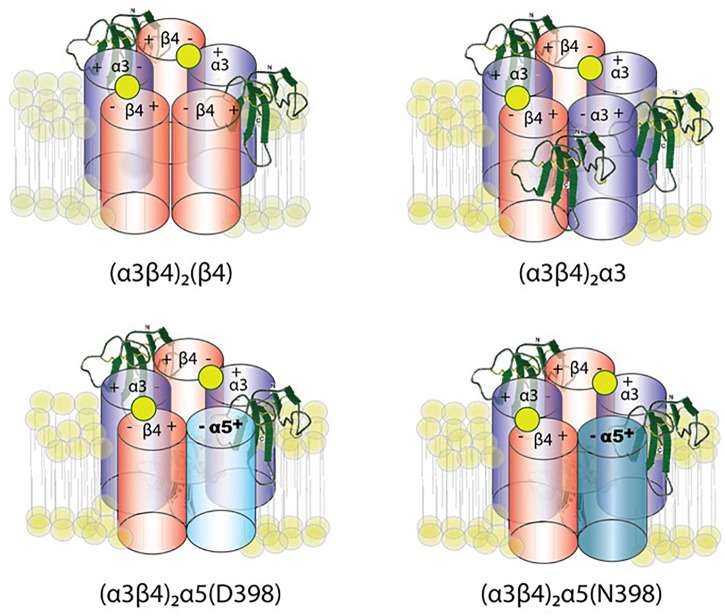
Working model of lynx1 modulation of α3β4- and α3β4α5-nAChR function. Lynx1, depicted in green, interacts with nAChRs that contain an α3(–) subunit interface (George et al., 2017). D398N mutation is associated with higher nicotine intake and relapse from quit attempts in humans. The N at position 398 is the risk allele.

George et al. (2017) constrained the number of variables using concatemeric nAChRs, in which five subunit cDNAs are fused into a single polypeptide, fixing the receptor stoichiometry. Co-expression of lynx1 with α3β4^∗^ nAChRs (^∗^-containing) suggests a role of lynx1 in altering channel opening, while previous studies have indicated that receptor number is altered only in some isoforms, depending on the subunit identity in the fifth position (George et al., 2017). Lynx1 reduces (α3)_2_(β4)_3_ cell-surface expression, whereas single-channel effects were primarily responsible for reducing (α3)_3_(β4)_2_ function by enhancing closed dwell times, and by reducing conductance and the number of long bursts. Reduced cell-surface expression and increased closed dwell times accounted for the reduction in (α3)_2_(β4)_2_α5 function mediated by lynx1. These data suggest a model of lynx binding in which the ratio of lynx1 to receptor depends on the receptor isoform (Figure 3). Along with expression studies of lynx1 in regions related to nicotine intake/aversion, these studies highlight the potential significance of lynx1 in nicotine addiction.

### Expression of Lynx1 in the CNS

Lynx1 is widely expressed throughout the CNS, although levels are relatively higher in the hippocampus, cortex, and cerebellum (Miwa et al., 1999; Thomsen et al., 2014). In addition to its extensive expression in the brain, lynx1 can be found in the retina (Maneu et al., 2010), lung (Fu et al., 2012), and spinal cord (Meyer, 2014). Previous studies have noted the interesting temporal expression profile of lynx1, which can be observed beginning around postnatal week 2 or 3 in mouse models (Miwa et al., 1999; Thomsen et al., 2014), exhibiting a close correlation with the critical period in the visual system. Changes in gene expression levels have been reported for lynx1 due to pharmacological and genetic treatment (Miwa et al., 2012).

### Lynx1 and Cortical Plasticity

Lynx1 acts as a negative regulator of plasticity in regions such as the hippocampus and cerebellum, and the interaction of lynx1 with nicotinic receptors has been shown to alter plasticity in the adult visual and auditory cortices (Morishita et al., 2010; Takesian et al., 2018). Due to the experience and nAChR-dependent plasticity that occurs in the visual cortex (V1), it is a well-defined model for understanding the age-dependent molecular effects of lynx1 interactions with nAChRs. Lynx1 mRNA and protein levels increase in V1 at the end of the critical period, thereby decreasing ocular dominance plasticity (Morishita et al., 2010; Sadahiro et al., 2016). For example, the effects of lynx1 on ocular plasticity are demonstrated by an increase in responsiveness in the visual cortex in adult lynx1-null mutant KO mice during arousal, and by the discovery of a juvenile form of plasticity mediated by an interaction between lynx1 and tissue plasminogen activator (Morishita et al., 2010; Bukhari et al., 2015). Furthermore, apical spine turnover in pyramidal neurons of layers 5 and 2/3 is doubled in the V1 of lynx1KO mice, and a selectively higher rate of loss is observed in the layer 5 of adults lacking lynx1 (Sajo et al., 2016). Taken together, these results suggest that lynx1 plays a role in the structural remodeling and spine dynamics required for plasticity in the visual cortex.

Recently, lynx1 has also been linked to a reduction in auditory plasticity via associations with the α4-containing nAChR in 5-HT_3A_R-positive cells (Takesian et al., 2018). In these studies, a nearly two-fold developmental increase in lynx1 expression in primary auditory cells was observed between P11 and P20, along with a decrease in nAChR sensitivity in 5-HT_3A_R cells. Furthermore, heightened nicotine sensitivity can be observed in lynx1KO mice, and such sensitivity can be attenuated using the α4 nAChR-specific antagonist DHβE (Takesian et al., 2018).

### Lynx1 and Associative Learning

Lynx1KO mice exhibit improvements in cognitive ability, for example, improved associative learning and memory behaviors (Miwa et al., 2006). Contextual memory (assessed using the Morris Water Maze, passive avoidance conditioning, and contextual fear conditioning) does not significantly differ between wild-type and lynx1KO mice, suggesting a specific role of lynx1 in associative learning. To further investigate phenotypic specificity, lynx1KO mice were tested for pain sensitivity (Nissen et al., 2018). Since the fear conditioning paradigm involves shock/tone pairing, this would serve as an important control test for the specificity of the learning phenotype. In one test of nociceptive signaling, the hot-plate test, lynx1KO mice exhibited no significant differences in performance when compared to their wild-type counterparts (Nissen et al., 2018). Indeed, nicotine administration reduced nociceptive behavior in lynx1KO mice (Nissen et al., 2018), suggesting a possible link between lynx1 and analgesia. Because dopamine levels may also influence associative learning/Pavlovian conditioning (Ikegami et al., 2014), researchers have investigated dopamine levels in lynx1KO mice (Parker et al., 2017). Such studies have revealed that lynx1KO mice exhibit dose-dependent decreases in dopamine levels when compared with their wild-type counterparts. Lastly, although lynx1 does seem to influence the function (but not assembly) of α6 nAChRs, as well as subsequent motor activity, such influence does not seem to extend to nicotine conditioned-place preference (Parker et al., 2017). The effects of lynx1 on α6^∗^ nAChRs seem subtle and are therefore unlikely to be a factor in associative learning as studied using these behavioral paradigms. Taken together, the effects of lynx1 are moderately specific to plasticity and associative learning.

### Role of Lynx1 in Alzheimer’s Disease (AD) Pathology

Alzheimer’s disease pathology is associated with an increase in soluble β-amyloid (Aβ), a peptide cleaved from the Alzheimer’s precursor protein (Inestrosa et al., 2013; Thomsen et al., 2016). Recent evidence suggests that lynx1 and Aβ_1–42_ compete for binding to nAChRs. In pull-down experiments from rat cortical extracts, ws-lynx1 pulled down all nAChR subunits tested (α3-7, β2, and β4), but the only subunits in which Aβ_1–42_ led to reduced lynx1/nAChR interactions were the α3, α4, α5, and α7 nAChR subunits. In contrast, the α6, β2, and β4 nAChR subunits were not sensitive to Aβ_1–42_ competition (Thomsen et al., 2016). Although the authors speculated that the interactions occurred at the neuronal cell surface because the incubation period was relatively short, the fact that interactions were insensitive to β subunits suggests a significant association of lynx1 with individual α subunits or non-pentameric receptors, since only α7 subunits from this list have been shown to form homopentamers. These interactions are likely to occur beneath the membrane surface, consistent with the reported interaction of lynx1 with nAChR dimers in the endoplasmic reticulum (Nichols et al., 2014). Conversely, when Aβ_1–42_ was used as the bait in pull-down experiments, it also pulled down all nAChRs tested, and lynx1 could compete at α7 and β2 subunits. Such findings are in accordance with the results of previous studies, which reported that Aβ_1–42_ can bind α7, α4β2, and α4α5β2 receptors (Dougherty et al., 2003; Wu et al., 2004; Lamb et al., 2005). These results indicate that lynx1 and Aβ_1–42_ bind at similar sites on nAChRs. Although Aβ_1–42_ is thought to bind at the orthosteric binding site, it is possible for two peptides to bind at different sites and still provide orthosteric hindrance to receptor binding. If the lynx1 and Aβ_1–42_ interactions are significant *in vivo*, lynx1 may exert protective effects against the pathological progression of AD (Thomsen et al., 2016), highlighting the need for further studies on the entire receptor complex (Thomsen and Mikkelsen, 2012). In support of this, the toxic effect of Aβ_1–42_ in primary neuronal culture was reduced in the presence of ws-lynx1 (Thomsen et al., 2016). This notion is further supported by previous studies that have demonstrated the protective effects of lynx1 in neuronal health (Miwa et al., 2006; Kobayashi et al., 2014). Importantly, in the AD mouse model (3xTg AD), a reduction of lynx1 protein of 10% in the cortex was observed (Thomsen et al., 2016), suggesting a link between lynx1 and AD pathology. Thus, further studies are required to elucidate the role of Aβ_1–42_ in normal and disease states (Kroker et al., 2013).

### Lynx2/Lypd1

The existence of multiple lynx family members is advantageous in that it allows for better spatial or temporal control over the cholinergic system since each exhibits varying expression patterns in different circuits. For instance, lynx1 and lynx2 exhibit complementary and non-overlapping expression patterns (e.g., hippocampal CA3 for lynx1 and CA1 for lynx2) (Miwa et al., 1999; Dessaud et al., 2006; Tekinay et al., 2009). The lynx2 gene expression also is enriched in well-described anxiety pathways, such as the amygdala and prefrontal cortex, which lends itself as a modulator of anxiety (Tekinay et al., 2009). Characteristic of the three-looped structure of the ly6/uPAR super family (Dessaud et al., 2006), the lynx2 protein binds to and suppresses the activity of nAChRs (Tekinay et al., 2009; Wu et al., 2015). *In vitro* immunoprecipitation experiments have demonstrated that lynx2 forms stable complexes with α7, α4β2, and α4β4 nAChRs (Tekinay et al., 2009; Wu et al., 2015). Co-expression of lynx2 and α4β2 leads to faster desensitization kinetics in response to acetylcholine (Tekinay et al., 2009) and a shift in the EC_50_ for acetylcholine (Tekinay et al., 2009), nicotine, and epibatidine (Wu et al., 2015) in the presence of lynx2. The presence of lynx2 also decreases the expression of α4β2 at the cell surface, suggesting a potential mechanism for the decreased response to agonists (Wu et al., 2015). There is also evidence that the lynx2 protein can blunt nicotine-induced upregulation of α4β2 (Wu et al., 2015). Lynx2-null mutant mice (lynx2KO) exhibit increased sensitivity to nicotine within the medial prefrontal cortex when compared to wild-type controls (Tekinay et al., 2009). These data suggest that ligand sensitivity is altered in the presence of lynx2, and that lynx2 also acts to inhibit the activity of nAChRs. The functional consequences of lynx2 removal include increased anxiety-like behaviors across several paradigms of anxiety, as well as reduced social interaction, suggesting that lynx2KO can be established as a robust mouse models of anxiety. Further studies into the removal of nAChR inhibition in lynx2KO mice are underway in order to determine the role of lynx2 in regulating specific nAChR subtypes *in vivo*. These studies underscore the importance of fine tuning the cholinergic system in a spatially controlled manner. The anxiety response includes a set of physiological changes that occur in response to a perceived threat. In the short term, this response is adaptive and helps defend against the threatening situation, but if the response is not regulated properly, it can lead to the development of anxiety disorders and significantly impact quality of life. Thus, lynx2 may play an important role in limiting or regulating the function of its cognate receptors to respond adaptively in circuits mediating anxiety-like behavior.

## Slurps

The Ly6/uPAR superfamily members discussed thus far are a part of the subfamily composed of membrane-bound GPI-anchored proteins. The other subfamily is composed of non-GPI anchored proteins (Adermann et al., 1999). The non-GPI anchored proteins are secreted and can exert a wide array of functions (Adermann et al., 1999; Fischer et al., 2001; Chimienti et al., 2003; Lyukmanova et al., 2014). SLURP-1 and SLURP-2 proteins represent two of these secreted, hormone-like cholinergic peptides of the ly6/uPAR superfamily (Adermann et al., 1999; Chimienti et al., 2003). SLURP proteins are found in the cytoplasm and function widely in non-neuronal cells, regulating growth and finely tuning the cell cycle (Moriwaki et al., 2009; Kessler et al., 2017). While SLURP proteins also largely target nAChRs, SLURP-2 can also interact with muscarinic receptors (Lyukmanova et al., 2016b). Most studies on SLURP function utilize recombinant or synthetic human SLURP proteins, as there are several methodological issues in obtaining the protein, such as an inability to obtain adequate amounts from natural sources, problems with production systems, and issues with protein folding (Lyukmanova et al., 2016a).

SLURP-1 is expressed in keratinocytes, where its presence modifies ACh signaling and epidermal homeostasis during cutaneous inflammation (Fischer et al., 2001; Chimienti et al., 2003); in primary sensory neurons in the dorsal horn of the spinal cord (Moriwaki et al., 2009); in airway epithelial cells, where its presence stimulates ciliary beating and decreases airway inflammation (Narumoto et al., 2010, 2013); and in corneal and ocular tissue (Swamynathan et al., 2015). Such studies have demonstrated the role of SLURP-1 in Mal de Meleda keratoderma (Fischer et al., 2001), nociception (Moriwaki et al., 2009), asthma (Narumoto et al., 2010, 2013), and pro-inflammatory conditions of the ocular surface (Swamynathan et al., 2015). Consistent with these studies, the SLURP-1KO mouse displays a palmoplantar keratoderma phenotype (Adeyo et al., 2014).

Studies using recombinant human SLURP-1 (rSLURP-1) are detailed in Table 1. Data for similar experimental conditions differed among some studies, likely due to the use of different recombinant versions. Taken together, the evidence suggests that SLURP-1 functions as an allosteric modulator of α7 nAChRs (Chimienti et al., 2003; Narumoto et al., 2013; Lyukmanova et al., 2014, 2016a, 2018; Durek et al., 2017). SLURP-1 can be immunoprecipitated and co-localized with α7 nAChRs (Lyukmanova et al., 2016a, 2018). Further studies have indicated that SLURP-1 does not compete with α-btx in oocytes or cause any changes in function on its own (i.e., without the presence of Ach as a ligand), suggesting an allosteric mode of action (Chimienti et al., 2003; Lyukmanova et al., 2016a; Durek et al., 2017). There is conflicting data on the role of SLURP-1, which has been shown to both increase and decrease the E_max_, and to alter and not alter the EC_50_ (Chimienti et al., 2003; Lyukmanova et al., 2016a; Durek et al., 2017).

**Table 1:** SLURP and nAChR studies.

SLURP	Functional Effect	Tag	Study
rSLURP-1 human	Increase in E_max_ Shift to left of EC_50_	C-terminal myc tag N-terminal HA tag	Chimienti et al., 2003
rSLURP-1	N/A	N-terminal Flag, C-terminal GPI	Moriwaki et al., 2009
rSLURP-1	Suppresses cytokine production	N-terminal MBP tag	Narumoto et al., 2013
rSLURP-1	Competes with α-bgtx at AChBPs but not at α7 nAChRs, decrease in E_max_, no change in EC_50_	N-terminal Met	Lyukmanova et al., 2016a
sSLURP-1	No α-btx competition at α7 nAChRs or AChBP	Chemical peptide synthesis of human SLURP-1	Durek et al., 2017
rSLURP-2	Reduces cell number, competes with epi and nic binding, partially competes with α-btx binding	Mature SLURP cleaved from His-SUMO	Arredondo et al., 2006
rSLURP-2 and rSLURP-1	Reduces cancer cell number (colorectal)	N-terminal Met	Lyukmanova et al., 2014
rSLURP-2	SLURP-2 increases cytokine production in NHEK cells	N-terminal FLAG epitope-tagged	Moriwaki et al., 2015
rSLURP-2	Decreases α7 currents except at low [SLURP-2]. Increases keratinocyte growth, binds multiple nAChRs	N-terminal Met	Lyukmanova et al., 2016a
rSLURP-2 and rSLURP-1	May decrease growth in five of six α7-expressing cell lines	N-terminal Met	Lyukmanova et al., 2018

Although such findings remain controversial, highlighting the need for characterization standards, further evidence suggests that SLURP-1 can also mediate inhibition of human α3β4, α4β4, and α3β2 nAChRs, as well as human and rat α9α10 nAChRs (Durek et al., 2017). The relevant data were derived from synthetically generated SLURP-1 (sSLURP-1), which exhibits similar structural yet different functional features when compared to rSLURP-1 (Durek et al., 2017). For instance, α-btx binding is not influenced by sSLURP-1 in torpedo membranes (muscle type nAChRs) or AChBP from either *A. Californica* or *L. Stagnalis*, whereas rSLURP-1 can compete with α-btx binding in torpedo membranes and AChBP from *L. Stagnalis*, although such findings were not observed at α7^∗^ nAChRs (Shulepko et al., 2013; Lyukmanova et al., 2016a). Functionally, sSLURP-1 exerts no effect on α7^∗^ nAChR responses in oocytes. When the α7 component was potentiated by a PAM in mammalian, chaperone co-transfected Neuro-2A cells, however, high doses of agonist were associated with alterations in the E_max_ of epibatidine. In such experiments, calcium-sensitive dyes were used to measure activity indirectly. In oocyte expression systems, sSLURP-1 exerts more robust inhibitory effects on α3β4 nAChRs, although effects can also be observed at α4β4 and α9α10 nAChRs. Based on the accumulated evidence, researchers have speculated that sSLURP-1 acts as a “silent” negative allosteric modulator, exerting its effects only when the nAChR is in the open state (Durek et al., 2017). Despite structural similarities (e.g., differences in the sequence of the N-terminal methionine only), rSLURP-1 and sSLURP-1 need to be tested side by side in the same assay in order to understand potential functional differences, highlighting the need for strict compound characterization standards if results are to be reproducible and transferable (Durek et al., 2017).

SLURP-2 is also an endogenous modulator of nAChRs with 10 cysteine residues and a 28–34% amino acid homology with Ly6 family members (Tsuji et al., 2003; Moriwaki et al., 2009; Peters et al., 2014). Historically, the LYNX1 gene was thought to give rise to both lynx1 and SLURP-2 due to alternative splicing, but recent evidence suggests that the genes are in close proximity but under the control of different transcription units (Moriwaki et al., 2015; Loughner et al., 2016). SLURP-2 differs from both SLURP-1 and lynx1 in that it has an overall negative charge (Lyukmanova et al., 2016b). Compared to SLURP-1, SLURP-2 (also studied via rSLURP2, see Table 1) immunoprecipitates with several nAChR subtypes (α3-α7, β2, and β4) (Lyukmanova et al., 2016b) and competes more efficiently with epibatidine than nicotine (Arredondo et al., 2006). SLURP-2 has been shown to interact with mAChRs in an heterologous CHO overexpression system (Lyukmanova et al., 2016b). Confirmation of pull down of native complexes in tissue or genetic confirmation to verify this finding will be informative. SLURP-2 is expressed in human epidermal and oral keratinocytes (Arredondo et al., 2006; Moriwaki et al., 2009, 2015), and in various tissues throughout the body, including epithelial tissue, the stomach, duodenum, esophagus, thymus, cervix, and uterus (Tsuji et al., 2003). Additional studies have indicated that SLURP-2 is upregulated in psoriatic lesional skin and in atopic dermatitis after a stress response, demonstrating its role in regulating stress-related cytokines in the skin (Tsuji et al., 2003; Peters et al., 2014; Moriwaki et al., 2015). The SLURP-2 KO mouse model also exhibits a palmoplantar skin disease phenotype (Allan et al., 2016). Both SLURP-1 and SLURP-2 can inhibit growth in cancer cell lines, suggesting an anticancer potential for these genes (Lyukmanova et al., 2014, 2018). However, overall, SLURP-2 acts in opposition to SLURP-1 to prevent apoptosis (Arredondo et al., 2006; Lyukmanova et al., 2016b).

## Prostate Stem Cell Antigen (Psca)

Prostate stem cell antigen is a GPI-anchored cell surface protein localized on lipid rafts (Reiter et al., 1998; Saeki et al., 2010). Although PSCA was originally characterized as a gene upregulated in prostate cancer, it is also expressed in the mammalian (Jensen et al., 2015; Ono et al., 2018) and avian brain (Hruska et al., 2009), and is up-regulated 70% in the cortex of AD patients (Jensen et al., 2015). Affinity purification studies have demonstrated that PSCA forms a stable complex with the α4 nAChR subunit but not the α7 subunit in the human temporal cortex (Jensen et al., 2015). However, its expression in the choroid plexus is worth noting since it is predicted to play a role in cell differentiation and proliferation in epithelial tissues (Ono et al., 2018). In one previous study, PSCA exerted an inhibitory effect on cell death induction in the chick ciliary ganglion via α7-containing nAChRs (Hruska et al., 2009). Furthermore, Jensen et al. (2015) revealed that PSCA decreases nicotine-induced ERK phosphorylation in PC12 cells and is dysregulated in the frontal cortex of patients with Alzheimer’s disease, highlighting the potential role of PSCA in pathologies that alter cognitive function.

## Lypd6

Another family member, lypd6, is highly expressed in the brain and spinal cord of mice and humans (Darvas et al., 2009; Zhang et al., 2010). Lypd6 regulates nicotinic receptor activity by enhancing Ca^+2^ currents through α/β-heterodimers in mice (Darvas et al., 2009). Lypd6 forms complexes with α3, α4, α5, α6, α7, β2, and β4 nAChR subunits and competes with α-btx for binding α7 subunits (Arvaniti et al., 2016). However, previous studies have also reported that blockade of α7 with α-btx and methyllycaconitine does not affect the modulation of nicotine-induced currents by lypd6 (Darvas et al., 2009). In contrast to the expression of lynx1 in parvalbumin cells, lypd6 is selectively expressed in somatostatin interneurons in cortical layers 5 and 6 of visual cortex region V1 (Darvas et al., 2009).

The function of lypd6 has been demonstrated in several model systems. For example, in PC12 cells, a soluble version of lypd6 completely inhibits nicotine-induced phosphorylation of ERK, which is an important pathway activated during plasticity induction. In addition, decreases in nicotine-induced currents can be observed when water-soluble lypd6 is applied to hippocampal slices (Arvaniti et al., 2016). In lypd6 overexpression studies, mice have demonstrated an increase in sensitivity to nicotine and behavior consistent with an increase in cholinergic tone, such as locomotor arousal, hypoalgesia, and pre-pulse inhibition of the acoustic startle response (Darvas et al., 2009). Furthermore, lypd6 KO mice exhibit decreased baseline levels of anxiety-like behavior in two-independent behavioral assessments (i.e., elevated plus maze and marble burying tests) (Arvaniti et al., 2018). Taken together, these results suggest that lypd6 plays a role in the negative modulation of nAChRs. Lypd6, however, also contains a Nxl motif, which allows it to bind LRP5/6, a member of the Wnt signaling pathway (Zhao et al., 2018), and therefore some of the phenotypes may be mediated by the Wnt coreceptor, low density lipoprotein receptor-related protein 6 LRP5/6.

## Lypd6b/Lypd7

Lypd6b, a member with large sequence similarity to lypd6, modulates nAChRs in a subtype-specific manner, and is expressed in glutamatergic neurons of the deep layer of the mammalian visual cortex (Demars and Morishita, 2014). Studies involving *Xenopus* oocytes have demonstrated that lypd6b displays isoform selectivity for inhibiting nAChR-mediated currents through α3β4 nAChRs; however, its presence does not alter α7 subtype-mediated currents (Ochoa et al., 2016). Furthermore, whole-cell recordings have revealed that lypd6b selectively reduces nAChR-mediated currents through α3β4, indicating that modulation of the receptor may occur at the α-α interface (Ochoa et al., 2016). The subtype selectivity and stoichiometry of lypd6b indicate that this prototoxin plays a highly specialized and complex role in nAChR modulation. Lypd7 was cloned from a human testis library, and is primarily expressed in the testes, prostate, stomach, and lung (Ni et al., 2009).

## Ly6h

In a previous *in vitro* study involving HEK cells expressing α7 nAChRs, a calcium-based FRET assay was used to determine the relative effect of a number of co-transfected ly6 proteins. To minimize receptor desensitization, the cells were treated with a positive allosteric modulator, PNU-120596, to allow for steady-state over kinetic measurements. The results indicated that there was a shift to the right of nicotine or epibatidine-evoked α7 responses when co-expressed with ly6h, similar to the shifts observed for lynx2 (Puddifoot et al., 2015). Ly6h also caused a reduction in the maximal response and surface expression of α7 nAChRs, suggesting a role for ly6h in receptor trafficking and gating. Prior to this study, the ly6 antigens, which are primarily expressed outside the brain, were not implicated in nAChR regulation. Ly6h has also been shown to influence α4β2 nAChRs in a similar FRET assay, although the effects of ly6h on nAChR trafficking were too pronounced to calculate a reliable EC_50_ in these studies (Wu et al., 2015). Interestingly, ly6h exerted no effects when co-expressed with α4β2 nAChRs in pull-down studies in HEKtsa or on desensitization rates of ACh-evoked responses in oocytes, in contrast to the significant effects observed for lynx2 (Tekinay et al., 2009). Differences in the heterologous systems or tagged proteins may have influenced these results.

In studies involving cells expressing α4β2 nAChRs, although there was a decrease in response to epibatidine by over 50% in the presence of lynx2 or ly6h, less pronounced effects were observed when to ly6e and ly6g6d were present. Such studies have revealed that ly6c1, ly6a, ly6c2, and lypd2 do not modulate α4β2 receptor activity. Furthermore, ly6a does not co-immunoprecipitate, change α4β2 expression at the surface, or interfere with nicotine-mediate up-regulation of α4β2 receptors (Wu et al., 2015).

## Ly6g6e

A relatively uncharacterized ly6 family member, ly6g6e, exhibits differences in function and mechanism from other members. Ly6g6e forms a complex with α4β2 nAChRs, and its presence potentiates rather than suppresses these receptors (Wu et al., 2015). Evidence suggests potentiation occurs due to direct modulation by ly6g6e at the cell surface. Cleaving the GPI anchor results in a loss of ly6g6e potentiation (Wu et al., 2015). Additional studies have indicated that ly6g6e can slow the desensitization of nAChRs (Wu et al., 2015). The potential mechanisms displayed by other family members, such as alterations in receptor expression at the cell surface or changes in nAChR ion selectivity, are unlikely, as ly6g6e does not alter the number of receptors at the membrane, and its effects persist in the absence of extracellular calcium (Wu et al., 2015). Ly6g6e expression has been detected in the hippocampus, cortical neurons, and in the midbrain (Wu et al., 2015). Based on its expression patterns and interactions with α4β2 nAChRs, which represent the main nAChRs contributing to nicotine addiction (Flores et al., 1992), ly6g6e may be involved in nicotine reward. Further studies are required to verify this possibility. The differential effects of ly6g6e on nAChRs demonstrate the diverse mechanisms of action present within the superfamily (Supplementary Table 1).

## Preferential Prostate and Testes Expression (Pate)-M and Pate-B

Preferential prostate and testes expression proteins conform to three-fingered protein/ly6/urokinase-type plasminogen activator receptor (uPAR) domains that shape three-fingered proteins in a manner similar to that of the prototoxins discussed above. Three human PATE genes (PATE-M, PATE-DJ, and PATE-B) have been identified and have demonstrated effects on nicotinic receptor function (Levitin et al., 2008). Two (hPATE-B and mPATE-C) enhance ACh-evoked net charge in oocytes expressing the homomeric α7 nAChR, while one (mPATE-P) reduces ACh-evoked net charge in oocytes expressing the α4β2 heteromeric nAChR (Levitin et al., 2008).

## Other Members of the Family

The ly6/uPAR superfamily continues to expand as more genes are characterized. Furthermore, interesting functions have been noted for members in invertebrate species, and 35 family members have been cloned from *Drosophila*, including *Coiled*, *Boudin* (Hijazi et al., 2009, 2011), and *Sleepless* (Koh et al., 2008). Interestingly, the protein bou is expressed in salivary glands and can incorporate into other cells, indicating a possible non-cell autonomous role.

## Prototoxin Binding Sites on nAchRs

Once the co-crystals of prototoxins and nAChRs have been identified, researchers should aim to determine the relevant binding sites, and whether these are associated with orthosteric or allosteric effects. The functional properties of prototoxins effects on nAChR function, such as desensitization and recovery from desensitization, although the effect of the GPI-anchored proteins on receptor trafficking, assembly, and stoichiometry may also account for these effects. Various reports have commented on the ability of prototoxins to compete on nAChRs with ligands that bind at the active site, such as nicotine, epibatidine, and α-btx (Lyukmanova et al., 2011; Durek et al., 2017; Arvaniti et al., 2018). Thus, differences in receptor types and the ligands used must be carefully considered (Xiu et al., 2009). An exogenously applied prototoxin that exerts modulatory effects on receptor function without competing with ligands provides support for allosterism.

While α-btx competition and nicotinic receptor upregulation studies have indicated that prototoxins may be involved in orthosteric interactions (Lyukmanova et al., 2013, 2016b; Wu et al., 2015), they are generally considered to be allosteric modulators (Ibanez-Tallon et al., 2002; Tsetlin, 2015; Lyukmanova et al., 2016a). Prototoxins that potentiate ligand-elicited currents can be regarded as positive allosteric modulators if they require acetylcholine or an agonist to open the receptor. Studies regarding the effect of lynx2 on nicotine-mediated up-regulation have suggested that lynx2 and nicotine operate at the same point in the receptor maturation pathway, potentially competing for binding at the ligand binding site. However, there are mechanisms that may allow for effects on receptor maturation that do not involve direct interaction at the same site on the receptor. Mutant cycle analyses have confirmed that actions at one part of the receptor can be communicated even across long distances from the receptor to the active site (Gleitsman et al., 2009).

There is a low-potency, high-efficacy, tertiary binding site on the α4: α4 interface of (α4)_3_(β2)_2_ nAChRs that can potentiate activation of the receptor by ACh (Indurthi et al., 2016). The presence of a third ligand at the two classical sites can lead to receptor transitions, detectable in both E_max_ and changes in the Hill slope. The authors contend that this is indicative of a pre-activational state similar to that of the benzodiazepine binding site on the GABA receptor (Indurthi et al., 2016). At present, it remains unknown how prototoxins interact with this third, low-potency site. Lyukmanova et al. (2016b) have suggested that SLURP-2 acts as a co-ligand for α7 nAChRs by priming it at low SLURP-2 concentrations. Although they postulate that SLURP-2 can work in concert with a single-bound ACh by binding at the orthosteric site, this only occurs at low SLURP-2 concentrations and is not accompanied by evident changes in Hill slope (Lyukmanova et al., 2016b). However, it would be interesting if a similar third binding site for agonists, with an ability to interact with prototoxins, were found to exist for α7 nAChRs. The differences in functional effects between rSLURP-1 and sSLURP-1 are most likely explained by the mode of synthesis vs. expression in *E. coli* vs. mammalian/insect cells and structural differences in nAChR types. With larger modulatory proteins, it is likely that both allosteric and orthosteric effects can be observed at nAChRs.

## The Case for *In Vivo* Tests of Prototoxin Binding in the Body and Brain

To our knowledge, there have been no unbiased investigations of prototoxin receptors to isolate every interacting partner of a prototoxin. This leaves open the possibility that prototoxins may bind to other classes of receptors outside of nAChRs *in vivo*. All tests of receptor interaction, whether positive (e.g., nAChRs (Ibanez-Tallon et al., 2002; Puddifoot et al., 2015), mAChRs (Lyukmanova et al., 2011, 2016b), LRP6 (Zhao et al., 2018), shaker potassium channels (Wu et al., 2016), or negative (e.g., glutamate, 5-HT3, etc.) have used either heterologously expressed receptor complexes, or have added the prototoxin to brain tissue. Only one study has isolated native complexes from the mammalian brain. In this study, β2^∗^ nAChR-containing receptors and lynx1 were identified using pull down studies from the mouse brain (Nissen et al., 2018), while ws-lynx1, SLURP-1 and SLURP-2 has been successfully mixed with human brain tissue to pull down multiple nAChR subtypes (Lyukmanova et al., 2016a,b; Thomsen et al., 2016). To support the *in vivo* interaction studies, a lynx1KO phenotype was ameliorated by crossing these mice into mutant mice null for α7 and β2 nAChR genes (Miwa et al., 2006). These *in vivo* studies provide an important biological context for the interactions observed within *in vitro* systems.

It should be noted that, even within brain tissues, many of these interactions may take place in the cytoplasm, and may therefore be independent of gating functions on the mature receptor pentamers at the cell surface. Receptor number can be reduced via the co-expression of prototoxin (Puddifoot et al., 2015; Wu et al., 2015; George et al., 2017), suggesting an effect on receptor retention or slowing of receptor maturation. Additional studies have indicated that the receptor complexes that escape to the cell surface may form complexes prototoxins (Nichols et al., 2014; Puddifoot et al., 2015; Wu et al., 2015), retaining their modulatory functions. Except in limited cases, however (Wu et al., 2015; George et al., 2017), prototoxins have been demonstrated to retain functional effects even when they have been cleaved from the GPI-anchor using PI-PLC, suggestive of stronger intracellular functions. Antibodies against these proteins that can discriminate cellular localization and surface vs. intracellular prototoxins are greatly needed to determine which of the multiple possible effects demonstrated thus far occur within the brain. Clearly, the interactions between prototoxins and their cognate receptors can be long-term, exerting varied (e.g., assembly and gating) and multiple (e.g., orthosteric and allosteric) effects throughout the life-time of a receptor (Supplementary Table 1).

## Promise of Prototoxin Studies for Therapeutic Development

Numerous studies have indicated that α4β2 and α7 nAChRs may represent targets for the treatment of various neurological and neuropsychiatric disorders (Graham et al., 2002; Picciotto and Zoli, 2008; Brunzell and McIntosh, 2012; Quik et al., 2012; Callahan et al., 2013; Nie et al., 2013; Wallace and Bertrand, 2013; Fan et al., 2015). Among the limitations in successfully targeting nAChRs are the sequence similarities amongst the individual subunits, as well as their widespread expression patterns. The elucidation of prototoxin function has potentially important consequences from a therapeutic perspective. The receptor/prototoxin complexes in specific tissues provide unique interfaces with more restricted expression profiles, allowing for more specific therapeutic modulation than the receptor alone. Furthermore, most prototoxins exhibit multiple binding specificities, which may be advantageous for regulating a multiplicity of nAChR subtypes in concert which govern a pathway, circuit, or biological function. Rather than targeting multiple nAChR subunits with multiple therapeutics, naturally evolving regulators of physiologically relevant functions, via allosteric interventions on nAChRs, may provide safer and more specific effects. Such an approach would enable a much more specific level of cholinergic regulation than can be achieved by targeting ACh levels or nicotinic receptor subtypes alone.

## Methods

### Molecular Phylogenetic Analysis

Reference protein sequences were obtained from GenBank or BLAST. Evolutionary analyses were conducted in MEGA7 (Molecular Evolutionary Genetics Analysis) (Kumar et al., 2016). Protein sequences obtained from GenBank were aligned using the MUSCLE algorithm within MEGA7 program. The evolutionary history was inferred by using the Maximum Likelihood method based on the Dayhoff matrix based model (Swartz and Dayhoff, 1979). Several substitution models were considered prior to selecting the Dayhoff model using MEGA7 model selection algorithms. Initial trees for the heuristic search were obtained automatically by applying Neighbor-Join and BioNJ algorithms to a matrix of pairwise distances estimated using a JTT model, and then selecting the topology with superior log likelihood value. The final bootstrap consensus tree inferred from 500 replicates (Felsenstein, 1985) was taken to represent the evolutionary history of the taxa analyzed. Branches corresponding to partitions reproduced in less than 50% bootstrap replicates were collapsed.

## Author Contributions

JM, KA, and KH wrote the manuscript. All authors have read and approved the manuscript. KA built tree and models for Figure 1.

## Conflict of Interest Statement

JM is founder of Ophidion, Inc. The remaining authors declare that the research was conducted in the absence of any commercial or financial relationships that could be construed as a potential conflict of interest. The handling Editor and reviewer VT declared their involvement as co-editors in the Research Topic, and confirm the absence of any other collaboration.

## Abbreviations

α-btx: alpha-bungarotoxin
5-HT_3A_R: 5-hydroxytryptamine receptors
Aβ: beta-amyloid
AChBP: acetylcholine binding protein
DHβE: dihydro-β-erythroidine
EC_50_: Effective concentration, 50%
E_max_: efficacy, maximal response
ERK: Extracellular Signal-regulated Kinase-1
GPI: glycosylphosphatidylinositol
HEK: human embryonic kidney
HS: high sensitivity
KO: knockout
LS: low sensitivity
ly6: lymphocyte antigen 6
Lynx: Ly6/neurotoxin
MEGA: Molecular Evolutionary Genetics Analysis
nAChR: nicotinic acetylcholine receptor
NMR: nuclear magnetic resonance
PATE: prostate and testes expression
PI-PLC: phosphoinositide phospholipase C
PSCA: prostate stem cell antigen
rSLURP: recombinant SLURP
SLURP1: secreted ly6/uPAR-related protein 1
SLURP2: secreted ly6/uPAR-related protein 2
sSLURP: synthetic SLURP
uPAR: urokinase-type plasminogen activator receptors
V1: visual cortex area 1
WTX: weak neurotoxin.
