# Supplementary material for: Lynx Prototoxins: Roles of Endogenous Mammalian Neurotoxin-Like Proteins in Modulating Nicotinic Acetylcholine Receptor Function to Influence Complex Biological Processes
Source: Front Pharmacol. 2019 Apr 30;10:343. doi: 10.3389/fphar.2019.00343 (PMC6502960; doi:10.3389/fphar.2019.00343)
Supplement: Supplementary file 1 [file Table_1.docx]

| Protein variant | assay | muscle nAChR | α7 nAChR | a4b2 nAChR | other | citation |  |
| --- | --- | --- | --- | --- | --- | --- | --- |
| ws-lynx1 | oocytes |  |  | increased peak ACh currents - presumably low [lynx1] | lynx1-Fc binding in cerebellum | Miwa, Neuron, 1999 |  |
| ws-lynx1 | α-btx binding | EC_50_ shift to the right | no effect on α-btx binding of α7 |  | AChBP IC_50_ 10 μM ws-lynx1 | Lyukmanova et al., 2011 |  |
|  | epibatidine binding | α-btx binding |  | no effect on epibatidine binding | small 25% increase in muscarinic binding |  |  |
|  | oocytes |  | decrease at low [ws-lynx1] 1 μM | no effect at low [ws-lynx1] 1 μM | CHO cells |  |  |
|  |  |  | increase at high [ws-lynx1] 10 μM | reduced ACh E_max_ at high [ws-lynx1] 10 μM | α3b2 no effect at low [ws-lynx1] |  |  |
|  |  |  | increased peak with multiple pulses | (no effect on desensitization) | α3b2 reduces E_max_ |  |  |
| ws-lynx1 | α-btx binding | torpedo | no effect on α7-glycine R binding |  | AChBP | Lyukmanova et al., 2013 |  |
|  |  | decreased binding | 1-30 μM ws-lynx1 |  | decreased α-btx |  |  |
|  | oocytes |  | decreased responses in α7-gly |  |  |  |  |
| ws-lynx1 | cell growth |  |  |  | no effect | Lyukmanova 2016, PlosOne |  |
| ws-lynx1 | ws-lynx1 BAC Tg- no GPI |  |  |  | enhanced motor learning - L7-secreted lynx1 | Miwa and Wlaz, 2012, PlosOne |  |
|  | ws-lynx1 BAC Tg - GPI |  |  |  | no effect on motor learning - L7- lynx1 |  |  |
| ws-lynx1 | pull down assay, h Temp Ctx |  | Binds α7 | binds α4, β2 | binds α3, α5, α6, β4 | Thomsen et al., Neurobiol Aging, 2016 |  |
|  | mouse Ctx,VTA, OB |  |  | binds α3, α4, α5, α6, α7, β2, β4 | (Ctx-AulB) |  |  |
|  | pERK stimulation, PC12 cells |  | Aβ reduced α7 | Aβ reduced α4, not β2 | α3β4 selective activation pERK  Aβ reduced α3, α5, α6, α7, not β4 |  |  |
|  | Aβ competition studies |  |  | AB1-42 pull down all  nAChRs, reduced  with ws-lynx1 for  α4, α7, β2 | |  |  |
|  | Aβ cytotoxicity |  | ws-lynx1 prevents Aβ cytotoxicity |  |  |  |  |
|  | AD 3xTG mice |  | 10% reduction in lynx1 levels |  |  |  |  |
| lynx1 (GPI) |  |  |  |  |  |  |  |
| lynx1 |  |  |  | modulatory with secreted lynx1 | toxin-like gene and structure | Miwa et al., Neuron, 1999 |  |
| lynx1 | immunostaining |  | co-expression in brain | co-expression in brain |  | Ibanez-Tallon et al., Neuron, 2002 |  |
|  | pull down |  | forms stable complexes | forms stable complexes | no binding to GRID |  |  |
|  | single-channel |  |  | higher conductance, fast inactivating openings | |  |  |
|  | oocytes |  |  | ACh EC_50_ shift to right, faster desensitization, slower recovery from desensitization | |  |  |
| lynx1 | lynx1KO mice |  | α7/lynx1KO | β2/lynx1KO | enhanced associative learning | Miwa et al., Neuron, 2006 |  |
|  | double KO mice |  | partially rescues neurodegeneration | partially rescues neurodegeneration |  |  |  |
| lynx1 | lynx1KO mice |  | extended critical period plasticity in visual cortex | MLA/DHβE cocktail blocks this phenotype | nicotine VEPs enhanced | Morishita et al., Science, 2010 |  |
| lynx1 | BAC Tg mice |  |  |  | no change in motor learning | Miwa and Walz, PlosOne, 2012 |  |
|  |  |  |  |  | (compared with s-lynx1) |  |  |
| lynx1 | FRET |  |  | Preferential binding  of α4:α4 over β2:β2  in the ER | | Nichols et al., JBC, 2014 |  |
|  | oocytes/n2A |  |  | PI-PLC removed lynx1  but does not alter  function | |  |  |
| lynx1 | lynx1KO mice, aging |  |  |  | age related loss of striatal neurons | Kobayashi et al., JMol Neurosci, 2014 |  |
| lynx1 | lynx1KO mice: V1 cortex analysis |  |  |  | sensory-dependent tPA elevation and dendritic spine reduction | Bukhari et al. 2015 |  |
| lynx1 | lynx1KO mice |  |  |  | Altered spine dynamics in visual cortex before and after MD | Sajo et al., 2016 |  |
| lynx1 | single channel |  |  |  | decreased surface expression of (α3)_2_(β4)_3_ | George et al., 2017 |  |
|  |  |  |  |  | (α3)_3_(β4)_2_ increased closed state, reduced conductance and bursts |  |  |
|  |  |  |  |  | (α3)_2_(β4)2α5 increased  closed state, reduced  PI-PLC sensitive cell  surface expression | |  |
| lynx1 | lynx1KO |  |  |  | reduced dopamine release | Parker et al., 2017, Nissen 2018 |  |
|  |  |  |  |  | lower sensitivity to thermal pain |  |  |
|  |  |  |  |  | reduced nicotine-stimulated motor activity |  |  |
| lynx1 | lynx1KO |  |  | reduced sensitivity | heightened nicotine sensitivity | Takesian et al., Nat. Neurosci, 2018 |  |
| lynx2 | oocytes |  |  | accelerates desensitization kinetics, EC_50_ shift to right |  | Tekinay et al., Proc Natl Acad, 2009 |  |
|  | Lynx2KO mice |  |  | Increased anxiety and decreased social interaction |  |  |  |
|  | pull down |  | lynx2 interacts with α7 nAChRs | lynx2 interacts with α4β2 nAChRs |  |  |  |
| lynx2 | Ca FRET assay |  | decreases E_max_ |  |  | Puddifoot et al., 2015 |  |
|  |  |  | shift in EC_50_ to the right, nicotine and epibatidine |  |  |  |  |
|  |  |  | Interaction between PNU and lynx2 |  |  |  |  |
|  | Pull down, HEK cells |  | reduced surface α7 nAChRs |  |  |  |  |
| lypd6 | Overexpression |  | Currents unaffected by α-btx or MLA |  | Increase locomotion in several assays, no change in anxiety-like behavior, increase in working memory in Y-maze, higher pre-pulse inhibition, anti-nociceptive responses in writhing test, nicotine dose response curve shifted to left in tail flick test | Darvas et al., 2009 |  |
|  |  |  |  |  | Increase Ca^2+^ component of nAChR currents, less nicotine-induced intracellular calcium |  |  |
| lypd6 | western blot |  |  |  | membrane bound protein, present in synaptosomes | Arvaniti et al., 2016 |  |
|  | extracts from human temporal cortex | Co‐purified with α7 | Co‐purified with α4 and β2 | Co‐purified with α3, α5, α6, and β4 nAChR | Arvaniti et al., 2016 |  |  |
|  | Nicotine induced  currents in CA1 | |  |  | lypd6 application reduced inward currents | Arvaniti et al., 2016 |  |
| lypd6 | lypd6KO mice |  |  |  | Decreased anxiety-like behavior (MB, EPM), no changes in cognitive performance (SAB) | Arvaniti et al., 2018 |  |
|  |  |  |  |  | Increased nicotine-induced inward currents in KO |  |  |
| lypd6b | oocytes |  | does not alter α7 nAChRs |  | reduced whole cell currents through α3β4 | Ochoa et al., 2016 |  |
| PSCA | western blot |  |  |  | Inhibits nicotine-induced phosphorylation of ERK | Jensen et al., 2015 |  |
|  | affinity purification |  | Does not isolate α7 |  | Isolates α4 |  |  |
|  | Tg-AD mice |  |  |  | PSCA levels are increased in frontal cortex |  |  |
| PSCA | RT-qPCR in human brain |  |  |  | expression in neural and choroid plexus in normal human brain and in brain tumors | Ono et al., 2018 |  |
| PATE-B etc. | oocytes |  | hPATE-B increase α7 response | mPATE-P reduced nAChR response in α4β2 |  | Levitin et al. 2008 |  |
| ly6H | Ca^2+^  FRET assay HEK |  | reduced E_max_ nicotine, epibatidine |  |  | Puddifoot et al., 2015 J Neurosci |  |
|  |  |  | shift in EC_50_ to the right, nicotine and epibatidine |  |  |  |  |
|  |  |  | no interaction between PNU and ly6H |  |  |  |  |
|  | primary hippocampal neurons |  | ly6H reduces surface  α7 nAChRs | |  |  |  |
|  |  |  | ly6H shRNA increases ACh currents |  |  |  |  |
|  | synaptic function |  | change in EPSC frequency, Ly6H shRNA |  |  |  |  |
| Ly6g6e | Ca^2+^  FRET assay in HEK cells |  |  | Increase in epibatidine E_max_, sensitive to PI-PLC |  | Wu et al., 2015 |  |
|  |  |  |  | does not influence  nicotine-induced  up-regulation | |  |  |
|  |  |  |  | slows desensitization | |  |  |
| other |  |  |  |  |  |  |  |
| ly6e, ly6g6d | Ca^2+^  FRET assay in HEK cells |  |  | reduced E_max_ epibatidine |  | Wu et al., 2015 |  |
